# CoRoPINN: Cognitive region optimized physics-informed neural networks

**DOI:** 10.1371/journal.pone.0358646

**Published:** 2026-09-21

**Authors:** Jingcong Li, Yu Liao, Ximeng Wang, Jie Deng

**Affiliations:** 1 College of Intelligent Systems Science and Engineering, Hubei Minzu University, Enshi, China; 2 College of Electronics and Information Engineering, Sichuan University, Chengdu, China; Srinath University, INDIA

## Abstract

The solution of partial differential equations (PDEs) remains a significant problem in scientific computing. Although physics-informed neural networks (PINNs) provide a mesh-free paradigm, pointwise constraints offer limited supervision between collocation points and uniform training can under-resolve regions with greater learning difficulty. To address these coupled limitations, this paper introduces **Co**gnitive **R**egion **O**ptimized **P**hysics-**I**nformed **N**eural **N**etworks (CoRoPINN). Rather than treating region optimization and cognitive learning as independent additions, CoRoPINN forms a closed feedback loop. Gradient-stability statistics calibrate a globally stable baseline radius, PDE residuals estimate pointwise learning difficulty, and the same difficulty signal jointly adjusts sample-specific neighborhoods and cognitive weights. This coupling expands continuous physical constraints around under-resolved collocation points while progressively reallocating optimization from easier to more difficult regions. We evaluate CoRoPINN on three one-dimensional PDEs with five backbone architectures, Poisson equations in two, three, and five dimensions, and Burgers2D. Across the five backbones, CoRoPINN achieves the lowest geometric-mean relative mean absolute error (rMAE) on all three one-dimensional benchmarks, reducing the aggregate error relative to the second-best method by 21.0%, 61.6%, and 52.9% on 1D-Reaction, 1D-Wave, and Convection, respectively. It also ranks first in both metrics for 2D and 5D Poisson, remains second in 3D, and attains the lowest rMAE and relative root mean square error (rMSE) among all four methods on Burgers2D. These results establish a clear overall accuracy advantage for CoRoPINN among the evaluated baselines.

## Introduction

Partial differential equations (PDEs) play a pivotal role in the mathematical modeling of complex phenomena across various disciplines, including physics, chemistry, biology, and financial engineering. The numerical solution of PDEs presents a fundamental challenge in scientific computing [1–3]. Traditional numerical methods, such as the finite element method [4], finite difference method [5], and finite volume method [6], have matured and become the standard approaches. However, these methods primarily rely on grid discretization, which poses significant challenges, including high computational costs and the curse of dimensionality when dealing with high-dimensional problems, complex geometric boundaries, or the resolution of inverse problems [7,8].

In recent years, PINNs have emerged as a mesh-free deep learning framework, offering a novel paradigm for solving PDEs [9]. PINNs embed governing equations, initial conditions, and boundary conditions into the neural network’s optimization objective as residual losses. By leveraging automatic differentiation techniques to compute partial derivatives, they encourage the neural network to satisfy physical laws while fitting data. This integration of physical prior knowledge with data-driven approaches demonstrates immense potential for solving direct and inverse problems in data-scarce scenarios [8,10].

The success of PINNs has prompted refinements along four complementary directions. The first direction focuses on network architectures and seeks to improve expressiveness or computational efficiency. The separable physics-informed neural network (SPINN) [11] processes coordinate dimensions independently to reduce the cost of high-dimensional PDEs, whereas the deep fuzzy physics-informed neural network (FPINN) [12] introduces fuzzy-logic layers to improve robustness to data uncertainty.

The second direction focuses on optimization strategies and seeks to make the PINN loss landscape easier to train. The gradient-enhanced physics-informed neural network (gPINN) [13] regularizes residual gradients, while the scale-invariant MultiAdam optimizer [14] balances the optimization rates of heterogeneous loss terms.

The third direction focuses on balancing the multiple objectives in the PINN loss. Neural tangent kernel (NTK) weighting [15] estimates the relative importance of individual loss terms, whereas self-adaptive loss-balanced PINN [16] uses convergence status and uncertainty to update the weights.

The fourth direction focuses on changing the training paradigm itself. The variational physics-informed neural network (vPINN) [17] expresses PDE constraints in integral form, residual-based adaptive refinement (RAR) [18] adds points where PDE residuals are large, and physics-informed generative adversarial networks (PI-GANs) [19] combine physical constraints with adversarial training.

**Fig 1:**
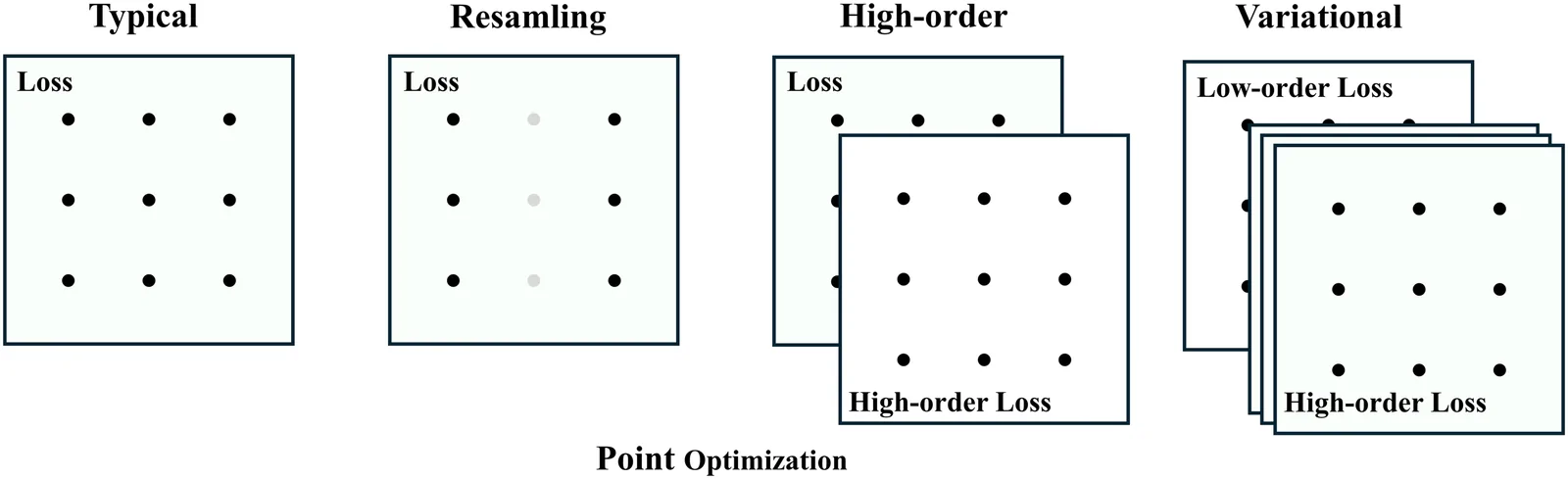
Point optimization PINNs.

Despite this progress, two interconnected limitations remain. First, conventional PINNs impose PDE constraints at finite, discrete points, although the target equations are defined over continuous domains [9,20]. This spatial mismatch weakens supervision between collocation points and impairs the representation of local fine-scale structures (Fig 1). Second, learning difficulty varies substantially across the domain, but conventional training assigns equal importance to all samples [21]. The resulting Unbalanced Prediction Problem (UPP) causes optimization to favor smooth or simple regions while under-resolving challenging structures such as boundary layers. Existing approaches address parts of these limitations, but they do not jointly coordinate continuous neighborhood constraints, pointwise difficulty, and training-stage progression within one feedback loop.

**Fig 2:**
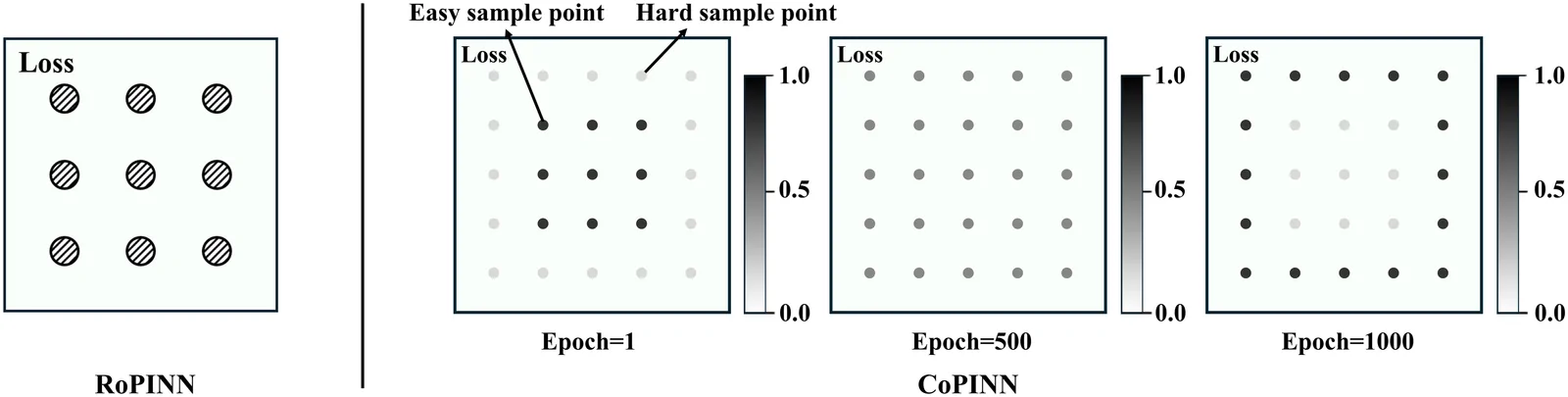
Training paradigm extension PINNs.

To address these challenges, two distinct approaches to expanding training paradigms have emerged (Fig 2). The region-optimized physics-informed neural network (RoPINN) [20] adopts a spatial continuity perspective, effectively enhancing local constraints and improving generalization capabilities by extending the optimization domain from discrete points to their continuous neighborhoods. Conversely, the cognitive physics-informed neural network (CoPINN) [21] focuses on temporal dynamics and introduces a self-paced learning mechanism that dynamically evaluates sample difficulty and adjusts learning weights, thereby effectively mitigating the UPP [22].

Existing adaptive PINN methods differ in both their optimization units and their adaptation targets. Residual-based adaptive refinement (RAR) reallocates discrete collocation points according to residual magnitude, but its optimization unit remains an isolated point [18]. RoPINN expands each point into a neighborhood and calibrates a shared region scale through gradient stability, but it does not explicitly model pointwise difficulty or training-stage progression [20]. CoPINN schedules pointwise loss weights from easy to hard, but it retains point-based constraints [21]. CoRoPINN differs by coupling a globally calibrated stable radius with residual-based sample difficulty, point-specific regions, and progressive cognitive weights in one feedback loop. This coupling links spatial continuity with temporal curriculum learning instead of treating region optimization and cognitive weighting as independent additions.

Accordingly, this paper introduces **Co**gnitive **R**egion **O**ptimized **P**hysics-**I**nformed **N**eural **N**etworks (CoRoPINN). Its central advantage targets PDEs whose learning difficulty is distributed unevenly over the solution domain. Difficulty-adaptive neighborhoods strengthen continuous supervision around under-resolved collocation points, while cognitive weights redirect optimization toward those regions as training progresses. The present benchmarks evaluate this capability through local fine-scale structures, sparse coverage in higher dimensions, and coupled nonlinear dynamics. Within this evaluated scope, the closed-loop coordination addresses limitations that neither region optimization nor pointwise cognitive weighting resolves alone.

The main contributions of this paper are as follows:

- We develop a cognition-guided region optimization mechanism that links global trust-region calibration, sample-specific residual difficulty, adaptive region sampling, and progressive cognitive weighting within one training loop.
- We formulate a dual radius update that first identifies a globally stable region scale and then adapts each sampling neighborhood to local learning difficulty.
- Across five backbone architectures, CoRoPINN achieves the lowest geometric-mean rMAE on all three one-dimensional benchmarks. Under the stated loss-ranking rule, it leads or ties in 37 of 45 reported metric cells and also attains the lowest rMAE and rMSE on Burgers2D.

## Preliminaries

### PDE definition

Consider a system of partial differential equations that incorporates equation constraints, boundary conditions, and initial conditions. This can be formalized as follows:


$$\begin{array}{c} \left\{ \begin{array}{c} ℱ(u)(x)=0,\;x\in \Omega ,\\ ℬ(u)(x)=0,\;x\in \partial \Omega ,\\ ℐ(u)(x)=0,\;x\in \Omega_{0}. \end{array}\right. \end{array}$$ (1)

where ℱ, ℬ, and ℐ denote the governing PDE, boundary-condition, and initial-condition operators, respectively [1]. The symbols Ω, ∂Ω, and $\Omega_{0}$ denote the spatiotemporal domain, its boundary, and the initial-time domain at *t* = 0, respectively. The target solution is $u:{\mathbb{R}}^{d+1}\to {\mathbb{R}}^{m}$, where *d* is the number of spatial dimensions and *m* is the number of solution components. The coordinate $x\in \Omega \subseteq {\mathbb{R}}^{d+1}$ combines spatial position and time, i.e., $x=\left(x_{1},x_{2},x_{3},...,x_{d},t\right)$, where *t* denotes time.

PINNs have been demonstrated to approximate the true solution through neural network parameterization, with the training objective being to minimize the loss function [9].

### Region optimization method

Region optimization is a training paradigm that extends the PINN optimization process from discrete sampling points to continuous neighborhoods. Conventional PINNs impose PDE constraints solely at finite sampling points, whereas region optimization theoretically reduces generalization error and enhances constraint satisfaction by applying continuous constraints within the neighborhood of each sampling point [20]. Region optimization comprises two iterative steps: Monte Carlo approximation and trust-region correction. The former effectively approximates the optimization objective, while the latter efficiently controls estimation error.

#### Region optimization definition.

For a set of sampling points *S*, with |*S*| denoting its cardinality, region optimization extends each point $x_{i}$ to its neighborhood $\Omega_{r}=[0,r{]}^{d+1}$, where *r* is the region radius and *d* denotes the spatial dimension. The region loss function can be defined as:


$$\begin{array}{c} {ℒ}_{r}^{\text{region }}\left(u_{\theta },S\right)=\frac{1}{\left|S\right|}\sum_{x\in S}{ℒ}_{r}^{\text{region }}\left(u_{\theta },x\right)=\frac{1}{\left|\Omega_{r}\right|\times |S|}\sum_{x\in S}\int_{\Omega_{r}}ℒ\left(u_{\theta },x+\xi \right)d\xi \end{array}$$ (2)

where $ℒ\left(u_{\theta },x\right)$ denotes the point optimization loss at *x*, $u_{\theta }$ is the neural network parameterized by θ, $\left|\Omega_{r}\right|$ is the measure (volume) of the extended neighborhood, and ξ is an offset within that neighborhood. This definition extends the original point constraint to a region integral constraint, requiring the network to satisfy the PDE not only at the sampling point but also maintain physical consistency within the surrounding neighborhood.

#### Monte Carlo approximation.

Since the region integral in Eq (2) cannot be computed directly, region optimization employs Monte Carlo approximation. At each iteration, an offset $\xi \sim 𝒰[0,r{]}^{\left(d+1\right)}$ is uniformly sampled from the neighborhood $\Omega_{r}$. The region loss gradient is approximated using the expected loss gradient at the sampled point [23], as follows:


$$\begin{array}{c} {𝔼}_{\xi \sim 𝒰\left(\Omega_{r}\right)}\left[\nabla_{\theta }ℒ\left(u_{\theta },x+\xi \right)\right]=\nabla_{\theta }{ℒ}_{r}^{\text{region }}\left(u_{\theta },x\right) \end{array}$$ (3)

Here, $𝒰(\Omega_{r})$ denotes the uniform distribution over $\Omega_{r}$, 𝔼 denotes expectation with respect to ξ, and $\nabla_{\theta }$ denotes the gradient with respect to the network parameters θ.

This approximation method has been demonstrated to efficiently implement region optimization while implicitly introducing a higher-order regularization effect [20]. The application of Taylor expansion analysis reveals that Monte Carlo sampling is equivalent to constraining the original loss and its first-order derivative terms. Such a constraint aids in suppressing unphysical oscillations in the solution, making it particularly suitable for PDE problems characterized by drastic variations.

#### Trust region calibration.

To prevent excessive sampling areas from causing optimization instability, region optimization employs a trust region calibration mechanism to adjust region size dynamically. The trust region is defined as the input domain area characterized by low gradient variance, where optimization is more stable and reliable [24]. The region radius *r* is dynamically adjusted based on gradient variance:


$$\begin{array}{c} r\propto \frac{1}{\left\|\sigma_{\xi \sim 𝒰\left(\Omega_{r}\right)}\left(\nabla_{\theta }ℒ\left(u_{\theta },x+\xi \right)\right)\right\|} \end{array}$$ (4)

where $\sigma_{\xi \sim 𝒰\left(\Omega_{r}\right)}(\cdot )$ denotes the gradient variance when uniformly sampling within the neighborhood $\Omega_{r}$. By approximating the variance through multiple consecutive iterations, the system adaptively adjusts the radius to balance generalization performance and optimization stability. In scenarios where the gradient variance is high, the region radius automatically shrinks to ensure convergence stability. Conversely, when optimization is stable, a larger region is permitted, thus enhancing generalization capability. This mechanism ensures stronger local constraints while maintaining training stability. The implementation-specific statistic, initialization, update frequency, and clipping controls used by CoRoPINN are specified in the later Trust region evaluation and Difficulty-based region adaptation subsections.

### Cognitive learning method

Cognitive learning is a machine learning training paradigm that emulates human learning processes. Its fundamental principle is the dynamic adjustment of learning strategies based on the difficulty level of learning materials [22]. In the domain of physical information, neural networks employ cognitive learning methods to facilitate a progressive training process, ranging from simplicity to complexity, by discerning variations in learning difficulty across different regions.

#### Difficulty evaluation in CoPINN.

The primary task for cognitive learning to achieve an easy-to-difficult progression involves accurately assessing the learning difficulty across various spatial locations. Difficulty levels are typically determined based on predefined heuristic rules, such as training loss or specific statistical properties of the data. In CoPINN, the difficulty metric for sampling points $x_{i}^{t}$ at the *t*-th iteration is defined by *t*he gradient norm of the PDE loss as:


$$\begin{array}{c} D_{i}^{t}={\left\|\frac{\partial {ℒ}_{pde}^{t}}{\partial x_{i}^{t}}\right\|}_{2} \end{array}$$ (5)

Here, *i* indexes a sampling point, *t* indexes the training iteration, ${ℒ}_{pde}^{t}$ is the PDE residual loss at iteration *t*, and $\|\cdot {\|}_{2}$ is the Euclidean norm. This metric dynamically reflects *t*he difficulty for the network to satisfy physical constraints at any given point. It does not rely on static, predefined difficulty measures but can continuously adjust its focus during training, emphasizing more challenging regions. A larger gradient norm indicates greater difficulty in satisfying the physical equations at that location. This phenomenon is often associated with boundary layers and other regions characterized by rapid changes [25,26].

#### Cognitive weight allocation.

Based on difficulty assessment results, the core task of cognitive learning is to dynamically assign evolving weights to different samples over time. In the early training phase, the differences in weights are maximal. At this stage, the network’s capacity to adapt to challenging regions is weak. The system assigns higher weights to simple samples to rapidly establish a stable, correct global solution structure. During the mid-training phase, weight differences reach a minimum. At this point, the model engages in comprehensive, balanced learning, consolidating existing knowledge while preliminarily exploring difficult areas to prevent premature convergence to local optima. In the late training phase, weight differences gradually widen again but in the opposite direction, shifting focus toward challenging samples. This concentrates optimization resources on overcoming residual local difficulties, achieving refined fitting [21]. The dynamic weight adjustment mechanism ensures a learning process that is both robust and efficient, effectively avoiding initial instability and late-stage underfitting.

## Method

### Problem analysis and motivation

PINNs are confronted with two fundamental challenges when attempting to solve PDEs: the local constraints of point optimization and the imbalance in spatial learning.

#### Limitations of point optimization.

Conventional PINNs minimize PDE residuals based on discrete sampling points, with the loss function defined as:


$$\begin{array}{c} ℒ\left(u_{\theta }\right)=\lambda_{pde}{ℒ}_{pde}+\lambda_{ic}{ℒ}_{ic}+\lambda_{bc}{ℒ}_{bc} \end{array}$$ (6)

Here, ${ℒ}_{pde}$, ${ℒ}_{ic}$, and ${ℒ}_{bc}$ denote the PDE residual, initial-condition, and boundary-condition losses, respectively, while $\lambda_{pde}$, $\lambda_{ic}$, and $\lambda_{bc}$ are their nonnegative weighting coefficients.

The PDE loss term is defined as:


$$\begin{array}{c} {ℒ}_{\text{pde }}=\frac{1}{N_{\Omega }}\sum_{i=1}^{N_{\Omega }}{\left\|ℱ\left(u_{\theta }\right)\left(x_{i}\right)\right\|}^{2} \end{array}$$ (7)

Here, $N_{\Omega }$ is the number of sampling points in Ω, ℱ is the PDE operator, and $x_{i}$ is a discrete point. This point-based optimization imposes constraints solely on a finite set of discrete points, which results in insufficient supervision between these sampling points. This issue is particularly pronounced for PDEs with highly variable solutions, where the local nature of point constraints can lead to significant generalization problems. It has been demonstrated that the network may exhibit minimal residuals at the sampling points, while simultaneously generating nonphysical oscillations in the intervals between these points [27–29].

#### Unbalanced prediction problem (UPP).

An analysis of error distributions during training reveals a severe spatial imbalance in PINNs when solving PDEs [21,29]. Notable disparities in learning difficulty have been observed across various spatial regions. In areas such as boundary regions and points of solution discontinuity, the residuals of PDEs are typically more than an order of magnitude higher than those in smooth internal regions. This phenomenon arises from drastic changes in physical quantities, leading to a highly uneven spatial distribution of prediction accuracy [15].

This imbalance stems from conventional PINN methods that apply identical optimization strategies across all training samples, neglecting the fundamental differences in physical properties and learning difficulty between regions. Since samples from simpler regions typically dominate the training process and gradient updates converge easily, uniform weight training strategies result in an overemphasis on these simpler regions. Consequently, this traps the model in local minima, hindering effective learning in more challenging regions that are critical to overall accuracy, thereby inducing the UPP.

#### Motivation analysis.

Region optimization has been shown to enhance generalization by expanding the constraint domain. However, this approach does not address imbalances in spatial learning. In contrast, cognitive learning can accommodate variations in difficulty through dynamic weight adjustments, yet it remains limited by the point optimization framework. These approaches are inherently complementary, enhancing PINNs from the perspectives of spatial continuity and temporal dynamics. The proposed CoRoPINN algorithm seamlessly integrates the strengths of both approaches. By concurrently adjusting the constraint scope and optimizing weights, the model adaptively allocates computational resources and constraint strength according to regional characteristics. This dual-adaptive mechanism facilitates customized treatment for regions with varying difficulty levels. Additionally, it establishes a dynamic equilibrium between local accuracy and global convergence, thus addressing the fundamental issues of locality and imbalance.

### Method framework

**Fig 3:**
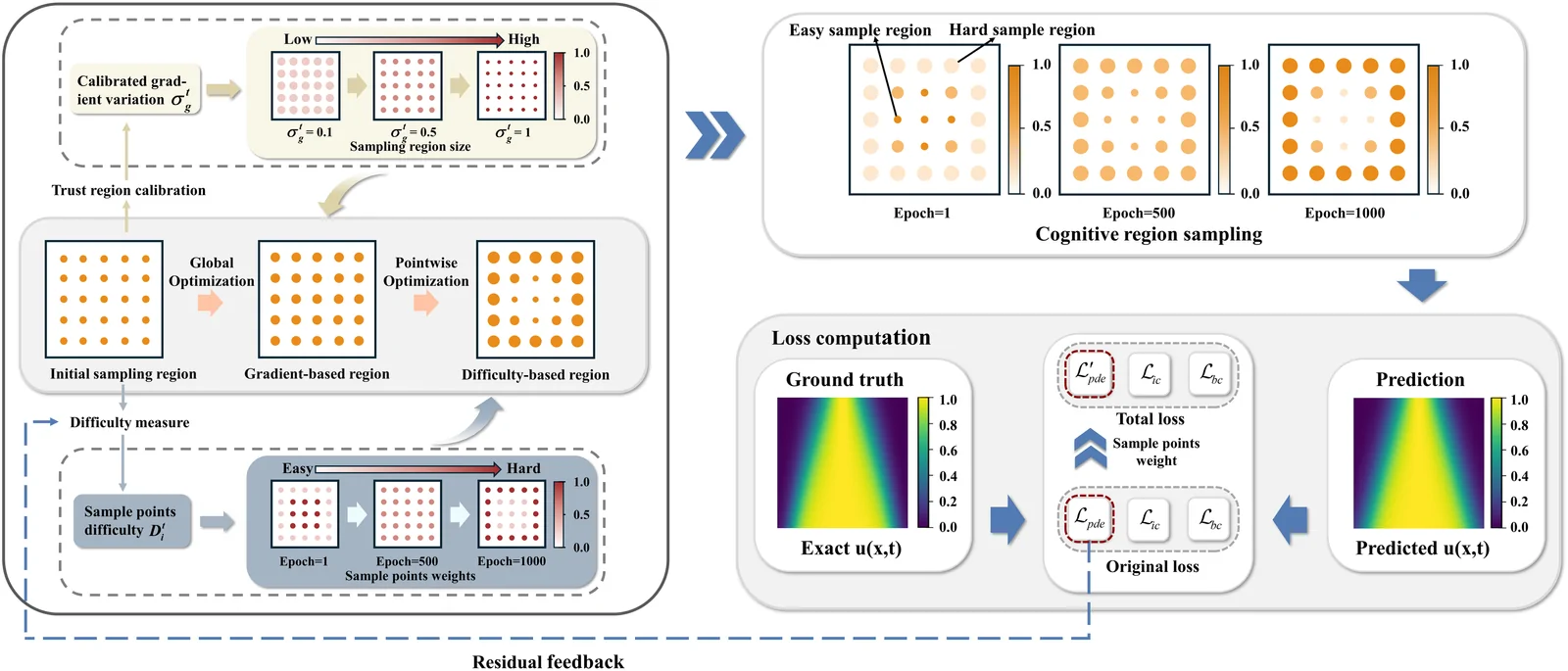
Overall workflow of CoRoPINN. The diagram shows the five-stage cognitive-regional optimization loop and the gradient-feedback path used to update trust-region calibration.

Fig 3 presents the end-to-end workflow of the tightly coupled cognitive-regional optimization loop. The workflow contains five stages. First, trust-region calibration evaluates optimization stability from the historical variation of network-parameter gradients and determines a stable baseline radius. Second, the PDE residual magnitude is used to evaluate the difficulty of each sample. Third, the raw and batch-normalized difficulty values adapt the pointwise sampling radius and cognitive weight, respectively. Fourth, Monte Carlo sampling is performed within each adaptive region, and the resulting residuals are combined into a cognitively weighted loss. Fifth, the limited-memory Broyden–Fletcher–Goldfarb–Shanno (L-BFGS) optimizer updates the network parameters [30], and the new gradient information is returned to the calibration module for the next iteration. These stages form a closed “calibration–evaluation–adaptation–sampling–feedback” loop.

### Theory analysis

#### Generalization error bound.

CoRoPINN effectively reduces generalization error by extending constraints from discrete points to continuous neighborhoods through region optimization. First, we define the model’s expected generalization error as [27]:


$$\begin{array}{c} \varepsilon_{gen}=\left|{𝔼}_{S,A}\left[ℒ\left(u_{A(S)},\Omega \right)-ℒ\left(u_{A(S)},S\right)\right]\right| \end{array}$$ (8)

where $\varepsilon_{gen}$ denotes the expected generalization error, and ${𝔼}_{S,A}$ denotes expectation over the sampled set *S* and any randomness in optimization algorithm *A*. The notation *A*(*S*) represents the parameters returned by *A* after training on *S*, and $u_{A(S)}$ is the corresponding model.

For positive constants *L* and β, assume that the point optimization loss ℒ is *L*-Lipschitz and β-smooth with respect to the model parameters. For arbitrary parameter vectors $\theta_{1}$ and $\theta_{2}$ and every sample point *x* in the global domain Ω, the following inequality holds [20]:


$$\begin{array}{c} \left\|ℒ\left(u_{\theta_{1}},x\right)-ℒ\left(u_{\theta_{2}},x\right)\right\|\leq L\left\|\theta_{1}-\theta_{2}\right\|,\left\|\nabla_{\theta }ℒ\left(u_{\theta_{1}},x\right)-\nabla_{\theta }ℒ\left(u_{\theta_{2}},x\right)\right\|\leq \beta \left\|\theta_{1}-\theta_{2}\right\|. \end{array}$$ (9)

Assuming ℒ is *L*-Lipschitz and β-smooth with respect to θ, performing *T* steps of stochastic gradient descent with step size $\alpha_{t}$ at step *t* yields the following conclusion [27,28]:

If ℒ is convex for θ and $\alpha_{t}\leq \frac{2}{\beta }$, then $\varepsilon_{\text{gen }}\leq \frac{2L^{2}}{\left|S\right|}\sum_{t=1}^{T}\alpha_{t}$ holds.

If ℒ is bounded by a constant *C* for all θ,x and is non-convex for θ with step size monotonically non-increasing $\left(\alpha_{t}\leq \frac{1}{\beta t},\;t\geq 1\right)$, then $\varepsilon_{\text{gen }}\leq \frac{C}{\left|S\right|}+\frac{2L^{2}(T-1)}{\beta (|S|-1)}$ holds.

Moreover, if ℒ is bounded and convex for all θ and *x*, and is *L*-Lipschitz and β-smooth with respect to the model parameters θ, then the region optimization loss function ${ℒ}_{r}^{\text{region }}$ is also bounded and convex for all θ and *x*, and is *L*-Lipschitz and β-smooth with respect to θ [20].

We assume ℒ is *L*-Lipschitz and β-smooth with respect to θ. Then, performing *T* steps of stochastic gradient descent on ${ℒ}_{r}^{\text{region }}$ with step size $\alpha_{t}$ at step *t* yields the following conclusion [20]:

If ℒ is convex for θ and $\alpha_{t}\leq \frac{2}{\beta }$, then $\varepsilon_{\text{gen }}\leq \left(1-\frac{\left|\Omega_{r}\right|}{\left|\Omega \right|}\right)\frac{2L^{2}}{\left|S\right|}\sum_{t=1}^{T}\alpha_{t}$ holds.

If ℒ is bounded by a constant *C* for all θ,x and is non-convex for θ with step size monotonically non-increasing $\left(\alpha_{t}\leq \frac{1}{\beta t},\;t\geq 1\right)$, then $\varepsilon_{\text{gen }}\leq \frac{C}{\left|S\right|}+\frac{2L^{2}(T-1)}{\beta (|S|-1)}-JL{\left(\frac{\left|\Omega_{r}\right|}{\left|\Omega \right|}\right)}^{2}$ holds, where *J* is a finite number determined by behavior in the early training phase.

It can be observed that, in comparison to point optimization, the generalization error bounds of region optimization introduce a negative term in both convex and non-convex scenarios, effectively reducing generalization error. This finding indicates that the region optimization paradigm offers a more consistent gradient optimization direction than point optimization at each iteration, thereby enhancing generalization performance.

#### Implicit regularization.

In solving PDEs, traditional point optimization imposes constraints on the values of derivative terms at specific points, resulting in significant locality concerns. In contrast, region optimization implicitly achieves high-order regularization effects within neighborhoods through Monte Carlo sampling. This approach is analogous to the concept of region constraints in Taylor expansion [13,20,31]:


$$\begin{array}{c} {𝔼}_{\xi \sim 𝒰\left(\Omega_{r}\right)}\left(\nabla_{\theta }ℒ\left(u_{\theta },x+\xi \right)\right)={𝔼}_{\xi \sim 𝒰\left(\Omega_{r}\right)}\left(\nabla_{\theta }ℒ\left(u_{\theta },x\right)+\nabla_{\theta }\left(\xi^{T}{ℒ}_{1}\left(u_{\theta },x\right)\right)+𝒪\left(\|\xi {\|}^{2}\right)\right) \end{array}$$ (10)

where $\Omega_{r}=[0,r{]}^{d+1},\;{ℒ}_{1}=\frac{\partial }{\partial x}ℒ\left(u_{\theta },x\right)$ defines the first-order loss function, and $𝒪\left(\|\xi {\|}^{2}\right)$ represents the truncation error of the Taylor expansion, encompassing all second-order and higher-order terms.

In cognitive region optimization, the constraint strength for higher-order derivatives is adjusted through difficulty adaptation. The globally calibrated baseline radius is expanded for points with larger residual difficulty and is bounded by the pointwise clipping rule defined below. This mechanism strengthens neighborhood constraints in challenging regions while retaining the stability imposed by trust-region calibration.

#### Convergence rate.

We give a first-order stationarity guarantee for a theoretical stochastic-gradient update of the time-varying CoRoPINN objective [20,32]. Let


$$\begin{array}{c} {𝒥}_{t}(\theta )=\frac{1}{N}\sum_{i=1}^{N}w_{i}^{t}{𝔼}_{\xi \sim 𝒰(\Omega_{r_{i}^{t}})}\left[\ell_{i}(\theta ;x_{i}+\xi )\right], \end{array}$$ (11)

where $w_{i}^{t}$ and $r_{i}^{t}$ are the cognitive weight and pointwise region radius at iteration *t*, respectively. Assume that (i) each ${𝒥}_{t}$ is lower bounded by ${𝒥}_{*}$ and is β-smooth; (ii) the weights and radii are bounded; (iii) the Mon*t*e Carlo gradient $g_{t}$ is conditionally unbiased, ${𝔼}_{t}[g_{t}]=\nabla {𝒥}_{t}(\theta_{t})$, with ${𝔼}_{t}\|g_{t}-\nabla {𝒥}_{t}(\theta_{t}){\|}^{2}\leq \sigma^{2}$; and (iv) a finite bound independent of *T* uniformly controls the cumulative objective drift:


$$\begin{array}{c} \delta_{t}=\sup_{\theta }\left|{𝒥}_{t+1}(\theta )-{𝒥}_{t}(\theta )\right|,\;\Delta_{T}=\sum_{t=0}^{T-1}\delta_{t}\leq \bar{\Delta }<\infty ,\;T\geq 1. \end{array}$$ (12)

**Proposition.** For the theoretical update $\theta_{t+1}=\theta_{t}-\eta g_{t}$ with $\eta =\alpha /\sqrt{T}$ and 0<α≤1/β, let *R* be sampled uniformly from {0,...,T−1}. Then


$$\begin{array}{c} 𝔼{\left\|\nabla {𝒥}_{R}(\theta_{R})\right\|}^{2}\leq \frac{2({𝒥}_{0}(\theta_{0})-{𝒥}_{*}+\bar{\Delta })/\alpha +\beta \alpha \sigma^{2}}{\sqrt{T}}=𝒪(T^{-1/2}). \end{array}$$ (13)

*Proof.* By β-smoothness, conditional unbiasedness, and the variance bound,


$$\begin{array}{c} {𝔼}_{t}[{𝒥}_{t}(\theta_{t+1})]\leq {𝒥}_{t}(\theta_{t})-\left(\eta -\frac{\beta \eta^{2}}{2}\right)\|\nabla {𝒥}_{t}(\theta_{t}){\|}^{2}+\frac{\beta \eta^{2}\sigma^{2}}{2}. \end{array}$$ (14)

Since η≤1/β, the gradient coefficient is at least η/2. Replacing ${𝒥}_{t}(\theta_{t+1})$ by ${𝒥}_{t+1}(\theta_{t+1})$ incurs at most $\delta_{t}$. Summing over *t*, applying $\Delta_{T}\leq \bar{\Delta }$ and ${𝒥}_{t}\geq {𝒥}_{*}$, and dividing by ηT/2 gives the result. This follows the RoPINN expected-descent structure [20], with the drift term accounting for CoRoPINN’s adaptive weights and radii.

### Cognitive region optimization

The cognitive region optimization framework constitutes the primary innovation of CoRoPINN. The integration of cognitive learning strategies with region optimization methods enables adaptive optimization across regions exhibiting varying degrees of difficulty.

#### Optimization objective.

The objective function of CoRoPINN integrates cognitive weight and region loss, as defined below:


$$\begin{array}{c} ℒ\left(u_{\theta },S\right)=\frac{1}{\left|S\right|}\sum_{x\in S}w_{i}^{\mathrm{cog}}(t)\cdot \left(\frac{1}{\left|M\right|}\sum_{m\in M}ℒ\left(u_{\theta },x+\xi_{m}\right)\right) \end{array}$$ (15)

where *i* indexes the point x∈S, *M* represents the set of Monte Carlo samples for each region, |*M*| is the number of such samples, and $\xi_{m}$ denotes the offset of the *m*-th sample. For the three one-dimensional experiments, define the training-progress factor and the easy/hard schedules as


$$\begin{array}{c} \tau (t)=\min\left(t/T_{\text{warmup}},1\right),\;v_{easy}(t)=1-0.7\tau (t),\;v_{hard}(t)=0.3+0.7\tau (t). \end{array}$$ (16)

Using the normalized residual difficulty ${\tilde{D}}_{i}^{t}$ defined below, the interpolated and implemented cognitive weights are [21]


$$\begin{array}{c} {\hat{w}}_{i}^{t}=v_{easy}(t)+\left[v_{hard}(t)-v_{easy}(t)\right]{\tilde{D}}_{i}^{t},\;w_{i}^{\mathrm{cog}}(t)=1+\beta_{c}\left({\hat{w}}_{i}^{t}-1\right). \end{array}$$ (17)

Thus, $v_{easy}(t)$ and $v_{hard}(t)$ are scalar base weights that respectively decrease and increase during warm-up. The coefficient $\beta_{c}$ scales the cognitive deviation from unit weight. The same coefficient also scales the pointwise region-radius correction below.

This objective function adjusts the contribution of samples with varying difficulty through cognitive weights, simultaneously enhancing local constraints through region sampling.

#### Cognitive difficulty evaluation in CoRoPINN.

Conventional PINN methods apply a uniform optimization strategy to all training samples, thus disregarding variations in learning complexity across regions. To explicitly capture such heterogeneity, CoRoPINN introduces a dynamic difficulty assessment mechanism based on PDE residuals [26].

Let $S_{t}=\{x_{i}^{t}{\}}_{i=1}^{\left|S_{t}\right|}$ denote the collocation batch at training step *t*. CoRoPINN measures the difficulty of $x_{i}^{t}$ using the absolu*t*e PDE residual:


$$\begin{array}{c} D_{i}^{t}=\left|{ℛ}_{u_{\theta^{t}}}\left(x_{i}^{t}\right)\right|, \end{array}$$ (18)

where $\theta^{t}$ denotes the network parameters and ${ℛ}_{u_{\theta^{t}}}$ denotes the PDE residual operator evaluated at step *t*. Thus, $D_{i}^{t}$ is the residual magnitude rather than an input-gradient norm. A larger value indicates *t*hat the current network violates the governing equation more strongly at that location.

For cognitive weighting, the residual magnitudes are normalized within each batch:


$$\begin{array}{c} {\tilde{D}}_{i}^{t}=\frac{D_{i}^{t}-D_{\min}^{t}}{D_{\max}^{t}-D_{\min}^{t}+\varepsilon_{D}},\;\varepsilon_{D}=10^{-8}, \end{array}$$ (19)

where $D_{\min}^{t}=\min_{i}D_{i}^{t}$ and $D_{\max}^{t}=\max_{i}D_{i}^{t}$. The stabilizer $\varepsilon_{D}$ avoids division by zero, and ${\tilde{D}}_{i}^{t}\in [0,1]$ provides a common scale for the cognitive weights.

The implementation also records the batch-average difficulty ${\overset{―}{D}}^{\;t}=|S_{t}{|}^{-1}\sum_{i}D_{i}^{t}$. For the most recent $N_{d}=10$ evaluations, the linear trend is obtained by least-squares fitting:


$$\begin{array}{c} (a_{t},b_{t})=\underset{a,b}{\mathrm{argmin}}\sum_{q=1}^{N_{d}}{\left[{\overset{―}{D}}^{\;t-N_{d}+q}-(aq+b)\right]}^{2}. \end{array}$$ (20)

Here, *q* indexes the $N_{d}$ retained evaluations, while $a_{t}$ and $b_{t}$ are the fitted slope and intercept, respectively. The sign of $a_{t}$ indicates whether the average residual difficulty is rising or falling. This trend is used as a bounded correction to the global trust-region statistic defined below.

#### Trust region evaluation.

To prevent excessive regions from destabilizing optimization, CoRoPINN first determines a globally stable baseline radius. Let $K_{t}$ be the number of optimizer closure evaluations at step *t*, and let *P* be the number of fla*tt*ened trainable parameters. The step gradient is


$$\begin{array}{c} g^{t}=\frac{1}{K_{t}}\sum_{k=1}^{K_{t}}\nabla_{\theta }{ℒ}_{CoRo}^{t,k}\in {\mathbb{R}}^{P}. \end{array}$$ (21)

Here, ${ℒ}_{CoRo}^{t,k}$ is the full closure-*k* loss at outer step *t* (weighted PDE, plus applicable boundary/initial terms), and $g^{t}$ is *t*he mean flattened closure gradient. For the history window ${ℋ}_{t}=\{\max(0,t-N_{g}+1),...,t\}$, the implementation calculates the mean coefficient of gradient variation:


$$\begin{array}{c} c_{g}^{t}=\frac{1}{P}\sum_{p=1}^{P}\frac{{\mathrm{Std}}_{q\in {ℋ}_{t}}(g_{p}^{q})}{{\mathrm{Mean}}_{q\in {ℋ}_{t}}\left(|g_{p}^{q}|\right)+\varepsilon_{g}},\;\varepsilon_{g}=10^{-6}, \end{array}$$ (22)

where $N_{g}$ is the retained gradient-history length, *p* indexes a flattened parameter component, Std and Mean denote the standard deviation and mean over ${ℋ}_{t}$, and $\varepsilon_{g}$ is a numerical stabilizer. The difficulty-trend correction and calibrated statistic are


$$\begin{array}{c} c_{D}^{t}=\left\{ \begin{array}{cc} 1+\eta \;\mathrm{sgn}(a_{t}), & N_{d}\text{ difficulty evaluations are available},\\ 1, & \text{otherwise}, \end{array}\right. \end{array}$$ (23)

$$\begin{array}{c} \sigma_{g}^{t}=c_{g}^{t}c_{D}^{t},\;\eta =0.1. \end{array}$$ (24)

Here, $c_{D}^{t}$ is the difficulty-trend correction, η=0.1 is its bounded amplitude, sgn is the sign function, and $\sigma_{g}^{t}$ is the calibrated gradient-variation statistic. If $\sigma_{g}^{t}=0$, it is set to 1 for numerical stability, as in the implementation. The statistic computed after step *t* calibrates the baseline radius used at step *t* + 1:


$$\begin{array}{c} {\overset{―}{r}}_{t+1}=\mathrm{clip}\left(\frac{r_{0}}{\sigma_{g}^{t}},0,r_{\max}\right),\;r_{\max}=0.01, \end{array}$$ (25)

where *r*_0_ is the user-specified initial region size and clip restricts its first argument to the stated lower and upper bounds. The first step uses the initialized calibration value $\sigma_{g}^{\left(-1\right)}=1$. A larger gradient variation therefore contracts the baseline region, whereas a stable gradient history permits a larger region within the stated bound.

#### Difficulty-based region adaptation.

After global calibration, CoRoPINN adapts the baseline radius to each collocation point using its raw residual difficulty:


$$\begin{array}{c} r_{i}^{t}=\mathrm{clip}\left({\overset{―}{r}}_{t}\left(1+\beta_{c}D_{i}^{t}\right),0.5{\overset{―}{r}}_{t},2{\overset{―}{r}}_{t}\right), \end{array}$$ (26)

where $\beta_{c}$ is the cognitive difficulty coefficient used by the implementation. It is distinct from the smoothness constant β in the theoretical analysis. The same $\beta_{c}$ scales both the cognitive weight and the pointwise radius: the raw value $D_{i}^{t}$ controls the latter, whereas ${\tilde{D}}_{i}^{t}$ controls the former. For each point, *M* Monte Carlo offsets are sampled as


$$\begin{array}{c} \xi_{i,m}^{t}\sim 𝒰\left([0,r_{i}^{t}{]}^{d+1}\right),\;m=1,...,M, \end{array}$$ (27)

and the governing-equation loss is evaluated at $x_{i}^{t}+\xi_{i,m}^{t}$. The clipping interval prevents the pointwise correction from changing the calibrated baseline by more than a factor of two.

Together, these equations specify the update order. CoRoPINN begins step *t* with the baseline radius calibrated from the previous gradient history, evaluates the current residual difficulty, adapts each pointwise radius, samples its neighborhood, and updates the network. The resulting gradients and average difficulty are then stored to calibrate the radius for step *t* + 1. This “calibration–evaluation–adaptation–sampling–feedback” sequence couples global optimization stability with local learning difficulty without altering the underlying PDE loss.

At each outer L-BFGS step, ${\overset{―}{r}}_{t}$ remains fixed across all closure evaluations. Each closure recomputes $D_{i}^{t}$ and $r_{i}^{t}$ from the current network state. After the optimizer step, the closure gradients are averaged to obtain $g^{t}$, and the retained gradient history and difficulty trend are updated. The resulting statistic then calibrates ${\overset{―}{r}}_{t+1}$. Thus, global calibration occurs once per outer step, whereas pointwise adaptation follows the current residual during each closure evaluation.

#### Progressive learning strategy.

To mitigate the instability that may arise from prematurely concentrating on challenging samples during the initial stages of training [33], CoRoPINN employs a progressive learning strategy governed by τ(t) above, where *T*_warmup_ denotes the warm-up period. During the warm-up phase (*t* < *T*_warmup_), the model prioritizes learning from simpler samples to es*t*ablish a stable foundational solution structure. As training progresses to $t\geq T_{\text{warmup}}$, the system gradually shifts its focus toward more challenging samples, thereby facilitating a natural transition from easy to hard.

#### Cognitive region sampling.

Based on the above mechanism, CoRoPINN employs a stratified region sampling strategy. The system continuously evaluates sample difficulty and tracks its evolving trends. For regions demonstrating persistent escalation in difficulty, the system not only expands the sampling scope through adaptive mechanisms but also augments their contribution to the loss function via cognitive weighting. Conversely, in regions where difficulty is reduced, the system dynamically reduces resource allocation. This cognition-guided sampling strategy ensures that computational resources remain concentrated on areas most in need of improvement, thus enhancing prediction accuracy while maintaining training efficiency.

## Experiments

### Experimental setup

#### Benchmarks.

To comprehensively validate the effectiveness of CoRoPINN, we select three representative one-dimensional time-dependent problems, three multidimensional Poisson problems, and Burgers2D as benchmarks.

The 1D-Reaction problem describes nonlinear reaction dynamics, the 1D-Wave problem represents hyperbolic wave propagation, and the Convection problem tests high-frequency transport. Together, they expose distinct optimization difficulties, including established failure modes of conventional PINNs [20,29,34].

Burgers2D complements these problems with coupled nonlinear transport–diffusion dynamics in two spatial dimensions and time [35].

The Poisson benchmarks are second-order elliptic equations on $[-1,1{]}^{d}$ with the manufactured solution $u(x)=\prod_{k=1}^{d}\sin(\pi x_{k})$. Increasing the dimension from 2D to 5D makes a fixed number of randomly sampled collocation points progressively sparser in the domain. The Poisson-5D setting uses 5000 interior points and therefore provides an explicit higher-dimensional sparse-coverage test. Table 1 summarizes the dimensionality, differential order, and principal characteristic evaluated by each benchmark.

**Table 1:** Summary of the benchmark problems used in this study.

Benchmark	Dimension	Highest derivative	Evaluated characteristic
1D-Reaction [20]	1D+Time	First order	Nonlinear reaction; PINN failure mode [29]
1D-Wave [20]	1D+Time	Second order	Hyperbolic wave propagation
Convection [20]	1D+Time	First order	High-frequency transport; PINN failure mode [29]
Poisson-2D [35]	2D	Second order	Elliptic solution in two dimensions
Poisson-3D [35]	3D	Second order	Increased-dimensional sparse coverage
Poisson-5D [35]	5D	Second order	High-dimensional sparse coverage
Burgers2D [35]	2D+Time	Second order	Coupled nonlinear transport–diffusion dynamics

Dimension reports the independent spatial and temporal variables. Highest derivative reports the differential order of the governing equation.

#### Base models and baselines.

To validate the broad applicability of CoRoPINN, five distinct neural network architectures were employed as baseline models. These included the canonical PINN [9], the quadratic residual network (QRes) [36], FLS (a sinusoidal-space PINN) [37], PINNsFormer (a Transformer-based PINN framework) [38], and the Kolmogorov–Arnold network (KAN) [39]. Based on these network architectures, we conducted comparative experiments between CoRoPINN and five baseline methods. Vanilla PINN represents the conventional physics-informed neural network algorithm, employing uniform sampling and standard loss functions. gPINN [13] enhances gradient information based on variational principles. vPINN [17] transforms point constraints into integral constraints using a variational formulation. RoPINN implements a region optimization strategy, employing Monte Carlo region sampling to expand the constraint domain. CoPINN employs a cognitive learning mechanism to dynamically adjust sample weights, thereby enabling progressive training from easy to difficult. CoRoPINN, the method proposed in this paper, integrates region optimization with cognitive learning to achieve dual spatial and temporal adaptive optimization.

#### Training configuration.

Training data are generated within each solution domain from the governing equation, boundary conditions, and initial conditions. For the three one-dimensional time-dependent problems, a uniform 101×101 space–time grid provides residual, boundary, and initial points. The Poisson experiments use randomly sampled interior and boundary points. The Burgers2D experiment uses randomly sampled interior, initial-condition, and periodic-boundary points. Table 2 summarizes the backbone configurations used in the comparisons.

**Table 2:** Neural network architectures used in the experiments.

Backbone	Architecture	Activation	Model-specific settings
PINN [9]	3 hidden layers, 512 units each	Tanh	Fully connected
QRes [36]	2 QRes blocks, width 256	Sigmoid	Quadratic residual blocks
FLS [37]	3 hidden layers, 512 units each	Sine then Tanh	Sine activation in the first layer
PINNsFormer [38]	1 encoder layer and 1 decoder layer, embedding dimension 32	Trainable sine–cosine activation (WaveAct)	2 attention heads; two 256-unit hidden layers in each encoder/decoder feed-forward part; two 512-unit hidden layers in the output head
KAN [39]	[1,2,5] or [1,2,5]	sigmoid linear unit (SiLU) and cubic splines	Grid size 5; spline order 3

For PINNsFormer, 256 and 512 denote the numbers of units in each hidden layer, not the numbers of layers. For KAN, the width vectors list units from input to output: [1,2,5] uses one five-unit hidden layer for 1D-Reaction, whereas [1,2,5] uses two for 1D-Wave and Convection. The PINN used for Poisson-2D and Poisson-3D has three 512-unit hidden layers, while Poisson-5D uses five. All use Tanh activation. The Burgers2D benchmark uses the same three-hidden-layer, 512-unit Tanh PINN, with (*x*,*y*,*t*) as three inputs and (*u*,*v*) as two outputs.

All models are trained in PyTorch [40] for 1000 outer optimization epochs. Optimization uses L-BFGS with a Strong-Wolfe line search. The initial learning-rate parameter (step size) is 1.0, and subsequent step sizes are selected by the line search.

#### Evaluation metrics.

Model performance is evaluated using three complementary metrics. The “Loss” metric assesses adherence to the physical constraints and is calculated as a weighted sum of the PDE residual, boundary-condition, and initial-condition losses. Relative mean absolute error (rMAE) denotes the average absolute deviation between the predicted and true values, normalized to the range of the true solution. This metric is robust to outliers and readily interpretable. Relative root mean square error (rMSE) measures the second-order moment of the prediction errors, making it more sensitive to large errors and informative about prediction quality in critical regions [41,42].

### Results

#### Comparison with standard and adaptive PINN baselines.

In this section, we compare six PINN training methods across fifteen backbone–PDE configurations, comprising five base architectures and three PDE problems. In addition to Vanilla PINN, the comparison includes gPINN, vPINN, RoPINN, and CoPINN. RoPINN and CoPINN are the most direct adaptive baselines because they isolate region optimization and cognitive weighting, respectively. The resulting matrix assesses CoRoPINN’s relative gains across backbone architectures and PDEs.

Table 3 presents a comprehensive comparison of CoRoPINN versus baseline methods across multiple typical PDE problems. Lower values for the rMAE, rMSE, and Loss metrics indicate superior model performance. Furthermore, to clearly present the results of the model comparisons, the optimal model results under the same experimental configuration are displayed in **bold**, while the second-best model results are shown in *italics* with underlines. The “Promotion” row records the percentage improvement or degradation of CoRoPINN in performance metrics relative to the best-performing model among the baselines.

**Table 3:** Comparison between CoRoPINN and standard or adaptive PINN baselines under different base models.

Base Model	Objective	1D-Reaction			1D-Wave			Convection		
		Loss	rMAE	rMSE	Loss	rMAE	rMSE	Loss	rMAE	rMSE
PINN [9]	Vanilla	2.0e-1	0.982	0.981	1.9e-2	0.326	0.335	1.6e-2	0.778	0.840
	gPINN	2.0e-1	0.978	0.978	2.8e-2	0.399	0.399	3.1e-2	0.890	0.935
	vPINN	2.3e-1	0.985	0.982	7.3e-3	0.162	0.173	1.1e-2	0.663	0.743
	RoPINN	4.7e-5	0.056	0.095	1.5e-3	0.063	0.064	1.0e-2	0.635	0.720
	CoPINN	**1.0e-6**	*0.038*	*0.066*	*4.2e-4*	*0.039*	*0.040*	**2.3e-3**	*0.624*	*0.705*
	CoRoPINN	*1.1e-5*	**0.025**	**0.045**	**1.5e-4**	**0.014**	**0.015**	*9.4e-3*	**0.585**	**0.668**
	Promotion	77%	34%	32%	90%	64%	63%	6%	6%	5%
QRes [36]	Vanilla	2.0e-1	0.979	0.977	9.8e-2	0.523	0.515	4.2e-2	0.925	0.959
	gPINN	2.1e-2	0.984	0.984	1.3e-1	0.785	0.781	1.6e-1	1.111	1.222
	vPINN	2.2e-2	0.999	1.000	1.0e-1	0.709	0.721	5.5e-2	0.941	0.966
	RoPINN	9.0e-6	*0.007*	*0.013*	*1.7e-2*	*0.309*	*0.321*	*1.2e-2*	0.819	0.870
	CoPINN	**2.0e-6**	0.010	*0.013*	8.9e-2	0.497	0.487	**7.71e-4**	*0.739*	*0.807*
	CoRoPINN	*3.0e-6*	**0.006**	**0.012**	**4.0e-3**	**0.103**	**0.120**	*1.2e-2*	**0.706**	**0.779**
	Promotion	67%	14%	8%	76%	67%	63%	0%	4%	3%
FLS [37]	Vanilla	2.0e-1	0.984	0.985	3.6e-3	0.102	0.119	1.2e-2	0.674	0.771
	gPINN	2.0e-1	0.978	0.979	9.2e-2	0.500	0.489	3.8e-1	0.913	0.949
	vPINN	2.1e-1	1.000	0.994	2.1e-3	*0.069*	0.069	1.1e-2	0.688	0.765
	RoPINN	2.2e-5	0.022	0.039	*1.5e-4*	**0.016**	*0.017*	9.6e-4	0.173	0.197
	CoPINN	**0**	**0.009**	**0.020**	7.1e-3	0.170	0.179	*2.6e-5*	*0.026*	*0.029*
	CoRoPINN	*4.0e-6*	*0.018*	*0.035*	**1.2e-4**	**0.016**	**0.016**	**5.0e-6**	**0.002**	**0.003**
	Promotion	82%	−99%	−75%	20%	0%	6%	99%	92%	90%
PINNsFormer [38]	Vanilla	3.0e-6	0.015	0.030	1.4e-2	0.270	0.283	3.7e-5	0.023	0.027
	gPINN	1.5e-6	*0.009*	0.018	OOM	OOM	OOM	3.7e-2	0.914	0.950
	vPINN	1.6e-4	0.065	0.124	4.5e-2	0.411	0.400	5.1e-5	0.016	0.022
	RoPINN	*1.0e-6*	**0.007**	*0.017*	6.5e-3	0.165	0.172	**1.2e-5**	*0.005*	*0.006*
	CoPINN	**0**	0.011	0.022	**5.5e-4**	**0.041**	**0.043**	4.8e-5	0.013	0.015
	CoRoPINN	3.0e-6	**0.007**	**0.015**	*1.9e-3*	*0.050*	*0.052*	*1.9e-5*	**0.004**	**0.005**
	Promotion	−99%	0%	12%	71%	−22%	−21%	−58%	20%	17%
KAN [39]	Vanilla	7.3e-5	0.031	0.061	9.2e-2	0.499	0.489	5.8e-2	0.922	0.954
	gPINN	2.9e-4	0.030	0.061	2.6e-1	1.131	1.110	1.2e-1	1.006	1.041
	vPINN	2.1e-1	0.998	0.996	9.0e-2	0.498	0.487	2.5e-2	0.853	0.853
	RoPINN	4.9e-5	*0.026*	*0.051*	*9.6e-3*	*0.177*	*0.191*	2.2e-2	0.805	0.801
	CoPINN	**1.0e-6**	0.031	0.062	2.2e-2	0.354	0.360	**9.2e-3**	**0.617**	**0.697**
	CoRoPINN	*4.5e-5*	**0.019**	**0.039**	**5.3e-3**	**0.066**	**0.067**	*1.2e-2*	*0.672*	*0.746*
	Promotion	8%	27%	24%	45%	63%	65%	45%	−9%	−7%

OOM denotes out of memory. Promotion values below −99% are reported as −99% for display because relative degradation is unbounded as the baseline value approaches zero.

To compare the six methods across backbone architectures at a glance, we aggregate the positive rMAE values in Table 3 using the geometric mean


$$\begin{array}{c} {\mathrm{GM}}_{rMAE}=\exp\left(\frac{1}{n}\sum_{j=1}^{n}\ln({rMAE}_{j})\right). \end{array}$$ (28)

**Fig 4:**
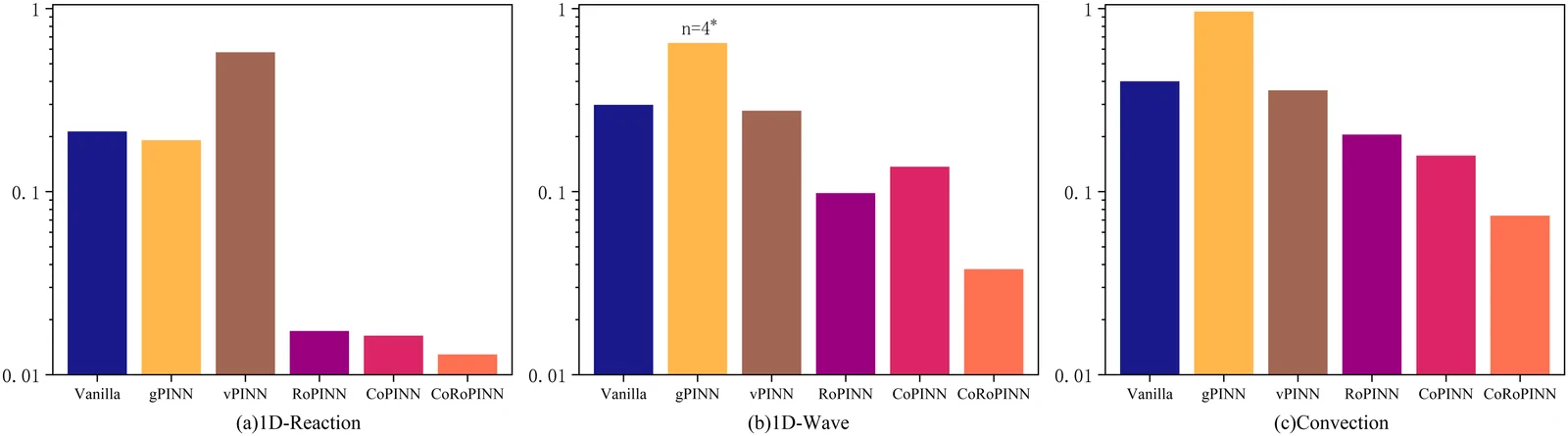
All-method comparison of geometric-mean rMAE across the five backbone architectures in Table 3. Lower values are better. The gPINN result for 1D-Wave uses the four successful configurations (*n* = 4) after excluding the PINNsFormer OOM entry. All other bars use *n* = 5. The rMAE values are shown on a logarithmic y-axis.

This ratio-scale summary avoids allowing a single result from a different error magnitude to dominate the cross-backbone comparison. Fig 4 shows that CoRoPINN achieves the lowest aggregate rMAE on all three problems: 0.012913 on 1D-Reaction, 0.037698 on 1D-Wave, and 0.074009 on Convection. Relative to the second-best aggregate in each problem, these values are lower by 21.0%, 61.6%, and 52.9%, respectively. The gPINN result for 1D-Wave is computed from four successful backbones because its PINNsFormer configuration was out of memory (OOM). No value is imputed for that configuration.

Under the PINN backbone, the direct adaptive-baseline comparison is consistent across the three PDEs. For 1D-Reaction, CoRoPINN obtains an rMAE of 0.025, compared with 0.056 for RoPINN and 0.038 for CoPINN. For 1D-Wave, the corresponding values are 0.014, 0.063, and 0.039. For Convection, they are 0.585, 0.635, and 0.624. These results demonstrate consistent gains under the canonical backbone across all three PDEs.

It is noteworthy that CoPINN, as a baseline method for cognitive learning, generally exhibits lower loss metrics. This phenomenon can be attributed to a global coefficient in its loss function that decays linearly toward zero, which can compress late-stage loss values without implying superior prediction accuracy. In contrast, CoRoPINN progressively redistributes relative emphasis between easy and difficult samples while retaining the bounded affine weight range $\left[1-0.7\beta_{c},1\right]$. This bounded rescaling avoids the global decay-to-zero behavior and reduces the resulting loss-scale bias. Because CoPINN loss values are therefore not directly comparable with those of the other methods, we exclude CoPINN from the Loss ranking. Under this stated rule, Table 3 shows that CoRoPINN is best or tied for best in 13 of 15 Loss cells and 24 of 30 rMAE/rMSE cells. CoRoPINN therefore leads or ties in 37 of the 45 reported metric cells across the evaluated settings.

#### Error distribution visualization.

This section evaluates the error distribution characteristics of CoRoPINN and baseline methods in solving PDEs. To achieve this, we systematically visualize the error distributions across four methods (PINN, RoPINN, CoPINN, CoRoPINN) for three problems (1D-Reaction, 1D-Wave, Convection). Our examination of the spatial distribution of absolute errors on both linear and logarithmic scales aims to reveal the differences in the methods’ capabilities to control local error maxima and enhance the overall smoothness of solutions. The central objective of this study is to determine whether CoRoPINN more effectively suppresses the UPP through the synergistic effects of region optimization and cognitive learning.

**Fig 5:**
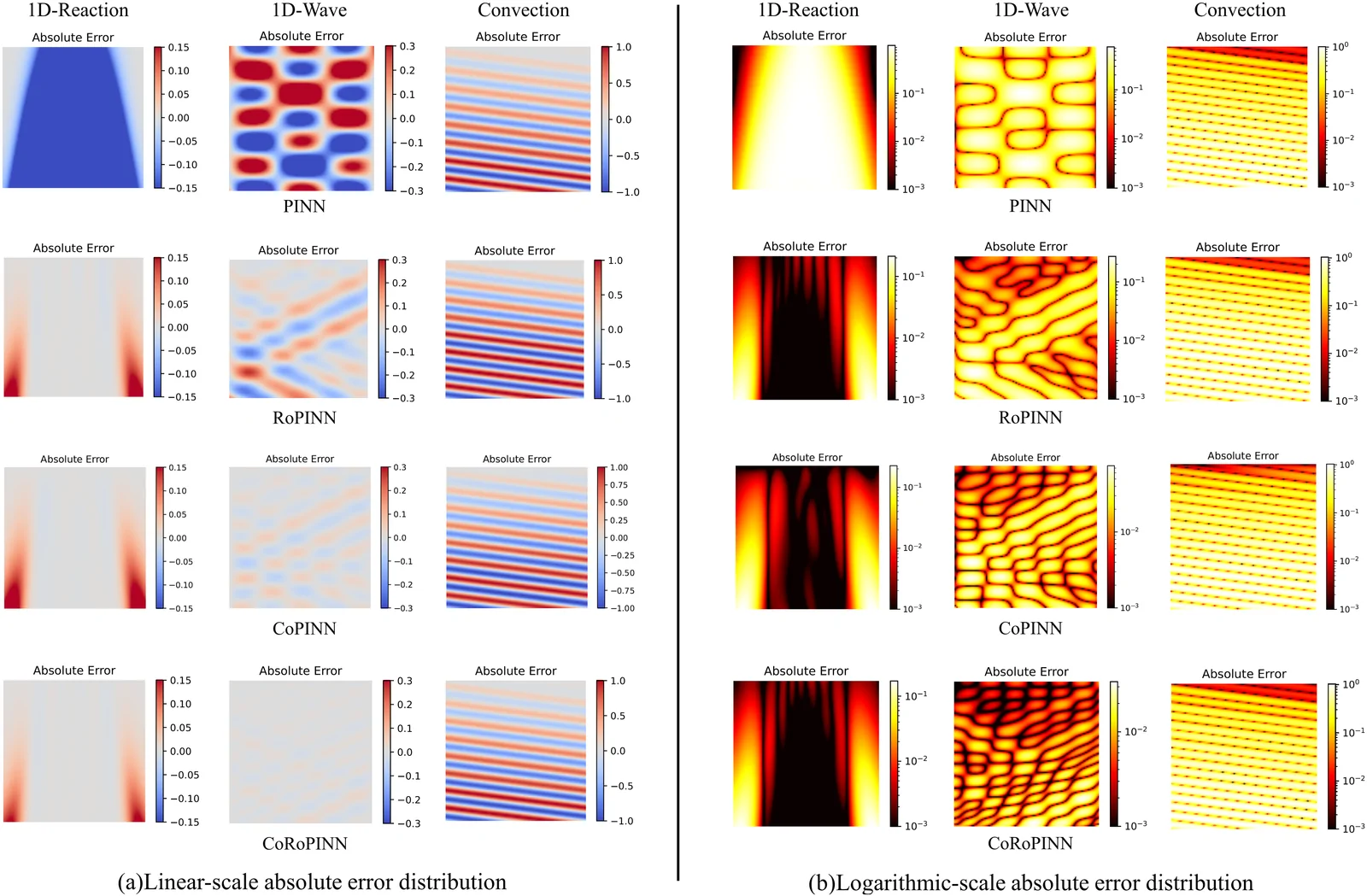
Absolute error distribution heat maps on different PDEs and diverse base models. (a) Results on the linear scale. (b) Results on the logarithmic scale.

Fig 5(a) presents a heatmap of absolute error distributions for the four methods on a linear scale, utilizing color gradients to visually represent the magnitude of errors. On this scale, the conventional PINN demonstrates the most significant fluctuations in error, characterized by substantial error ranges and notable clustering. RoPINN demonstrates distinct edge error clustering, manifesting as clear stripes in the boundary regions. CoPINN exhibits relatively uniform overall error distribution but still shows visible error fluctuations and localized high-error regions. In contrast, CoRoPINN demonstrates the most uniform error distribution, with the most consistent global color tone.

Fig 5(b) presents a replot of the spatial distribution of absolute error on a logarithmic scale. This scaling method enhances the contrast in regions with minimal error, thereby facilitating a more precise evaluation of subtle variations in approximating the exact solution. CoRoPINN exhibits superior spatial uniformity in error distribution and reduced overall error levels, notably mitigating error concentration in boundary areas.

The visualization of error distribution indicates that CoRoPINN exhibits superior spatial uniformity in both linear and logarithmic scales, providing qualitative support for the effectiveness of region optimization and cognitive learning mechanisms in improving error distribution.

#### Prediction results visualization.

**Fig 6:**
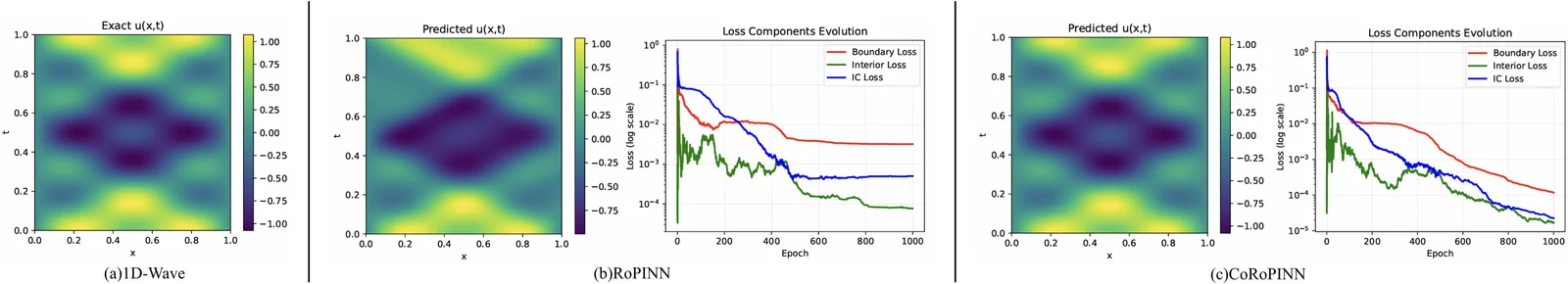
Comparison between CoRoPINN and RoPINN on the 1D-Wave equation using the PINN backbone. (a) Exact solution of the 1D-Wave equation. (b) RoPINN prediction and loss curves. (c) CoRoPINN prediction and loss curves.

To illustrate the performance of CoRoPINN, Fig 6 compares predictions obtained with the conventional PINN backbone on the 1D-Wave problem. The figure presents spatiotemporal heatmaps of the exact solution, RoPINN prediction, and CoRoPINN prediction, together with the loss curves for the two methods.

The visualization shows that the CoRoPINN prediction heatmap closely follows the exact solution and captures the propagation and boundary-reflection patterns of the wave equation. RoPINN reduces the limitations of point optimization through region optimization but retains visible prediction deviations. The loss curves provide a consistent quantitative comparison: CoRoPINN attains a 90% lower final loss than RoPINN, and all three loss components reach lower final values.

Notably, the loss curve of CoRoPINN exhibits a more stable descent trend, particularly during the mid-to-late training stages. Boundary loss and initial condition loss continue to decrease steadily, while RoPINN experiences significant stagnation in convergence during the same period. This phenomenon validates the effectiveness of incorporating cognitive learning mechanisms. By progressively shifting the focus of optimization from simple regions to challenging boundary areas, CoRoPINN avoids local optima traps during training and achieves comprehensive learning of critical regions such as boundaries.

#### Multidimensional PDE results.

To evaluate the generalization potential of CoRoPINN beyond the one-dimensional benchmarks, we include Poisson equations in 2D, 3D, and 5D. We further compare the four methods on Burgers2D using a shared PINN backbone and training protocol. The 5D case provides a direct higher-dimensional evaluation. The optimal result for each problem is highlighted in **bold**, while the second-best result is *italicized* and underlined.

As shown in Table 4, CoRoPINN demonstrates a clear overall advantage across the evaluated 2D, 3D, and 5D Poisson equations. In 2D, it ranks first in both metrics, reducing rMAE and rMSE by 29.0% and 41.2%, respectively, relative to the second-best CoPINN results. In 3D, CoRoPINN ranks second in both metrics and remains more accurate than Vanilla PINN and RoPINN. In the 5D case, it ranks first again and reduces rMAE by approximately 25% and 32% relative to RoPINN and CoPINN, respectively. Recovering the lead in 5D, where a fixed collocation budget leaves progressively sparser domain coverage, highlights CoRoPINN’s ability to use difficulty-adaptive neighborhoods to extract more information from limited collocation points. The coupled mechanism is therefore not tied to a single dimensional setting: it remains competitive in 3D and becomes most advantageous when sparse coverage makes isolated point constraints less effective.

**Table 4:** Results of CoRoPINN and comparison algorithms on multidimensional PDEs using the PINN backbone.

PDE	Dimension	Vanilla		RoPINN		CoPINN		CoRoPINN	
		rMAE	rMSE	rMAE	rMSE	rMAE	rMSE	rMAE	rMSE
Poisson [35]	2D	3.9e-3	5.3e-3	1.9e-3	2.8e-3	*1.0e-3*	*1.7e-3*	**7.1e-4**	**1.0e-3**
	3D	1.1e-3	1.2e-3	1.1e-3	1.2e-3	**4.6e-4**	**5.3e-4**	*5.2e-4*	*6.0e-4*
	5D	3.3e-3	4.2e-3	*2.0e-3*	*2.7e-3*	2.2e-3	3.2e-3	**1.5e-3**	**1.9e-3**
Burgers [35]	2D+Time	*0.487*	0.546	0.496	*0.542*	0.509	0.574	**0.481**	**0.531**

The multidimensional experiments include Poisson equations in 2D, 3D, and 5D, together with Burgers2D [35].

On Burgers2D, CoRoPINN attains the lowest rMAE (0.481) and rMSE (0.531) among the four methods. These values are 1.2% and 2.0% lower, respectively, than the corresponding second-best results. This extends the observed accuracy advantage to a coupled nonlinear transport–diffusion system and shows that the same core mechanism remains effective beyond scalar Poisson equations.

### Ablations

In this section, we validate the independent contributions and synergistic effects of the two core components–cognitive learning and region optimization–through systematic ablation studies conducted on three PDEs. The primary focus of these studies is to assess model performance and efficiency.

#### Performance analysis.

**Fig 7:**
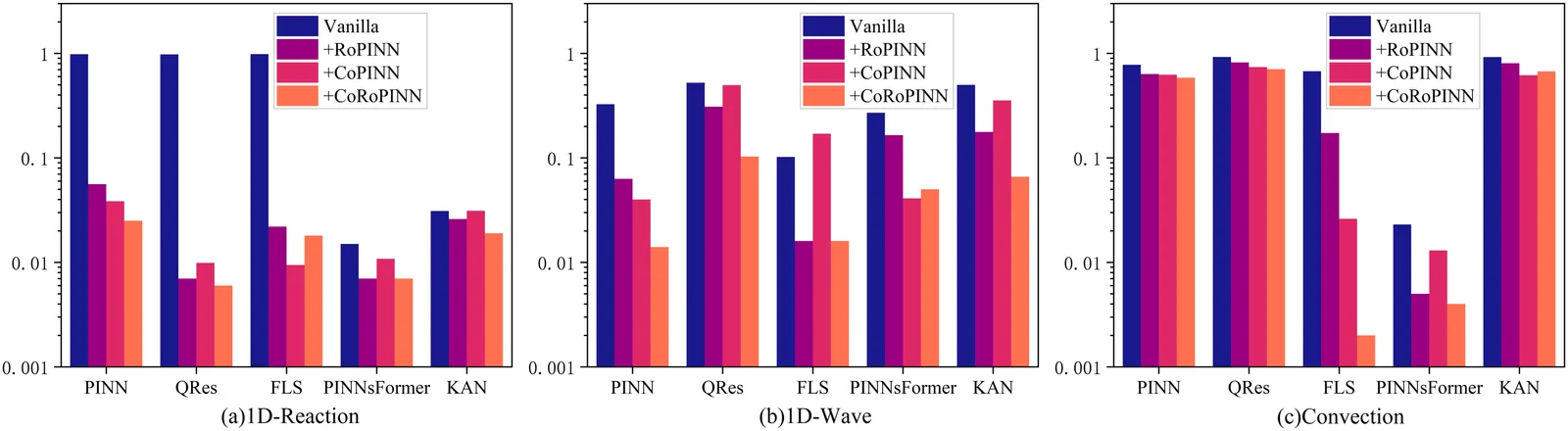
Accuracy ablation study of CoRoPINN on different PDEs and diverse base models. rMAE is recorded. The rMAE values are shown on a logarithmic y-axis.

As illustrated in Fig 7, we present a comparative analysis of rMAE across five distinct base network architectures. The conventional PINN approach is represented by Vanilla, while RoPINN and CoPINN denote methods employing region optimization and cognitive learning, respectively. CoRoPINN represents the comprehensive method proposed in this paper.

Overall, CoRoPINN achieves optimal or near-optimal performance across all test configurations. When compared to the Vanilla baseline, the introduction of either region optimization or cognitive learning individually yields significant improvements. This ablation study validates our core hypothesis: region optimization and cognitive learning enhance PINNs from the perspectives of spatial continuity and temporal dynamics, respectively, demonstrating their inherent complementarity.

#### Efficiency analysis.

Although CoRoPINN achieves favorable prediction accuracy, cognitive evaluation and region sampling introduce additional computation. Table 5 reports both rMAE and wall-clock training time in seconds per 100 epochs for the four methods. All entries use the same PINN backbone, collocation data, optimizer family, and evaluation interval within each PDE.

**Table 5:** Accuracy and wall-clock training-time comparison using the PINN backbone.

Method	1D-Reaction	Time (s)	1D-Wave	Time (s)	Convection	Time (s)
PINN [9]	0.982	10.5	0.326	19.2	0.778	48.3
PINN+RoPINN [20]	0.056	23.2	0.063	50.6	0.635	54.2
PINN+CoPINN [21]	*0.038*	22.9	*0.039*	90.6	*0.624*	55.1
PINN+CoRoPINN (ours)	**0.025**	26.0	**0.014**	78.4	**0.585**	55.8

Accuracy columns report rMAE. Time is wall-clock seconds per 100 training epochs under a common experimental setup for each PDE.

The results show that CoRoPINN requires more training time than standard PINN. On 1D-Reaction, PINN is the fastest method at 10.5 s per 100 epochs but has an rMAE of 0.982. CoRoPINN attains an rMAE of 0.025 at 26.0 s, compared with 0.056 at 23.2 s for RoPINN and 0.038 at 22.9 s for CoPINN. In this setting, CoRoPINN attains the lowest rMAE among the four methods at the cost of a moderate increase in training time relative to the adaptive baselines.

The computational trade-off also depends on the PDE and comparator. On 1D-Wave, CoRoPINN requires 78.4 s per 100 epochs, compared with 90.6 s for CoPINN, while attaining a lower rMAE of 0.014 versus 0.039. On Convection, the four methods have closer training times, whereas CoRoPINN records the lowest rMAE among the compared methods.

Overall, the table shows that the measured accuracy–cost balance varies across the three evaluated PDEs. The additional operations therefore remain a practical limitation that should be considered when CoRoPINN is transferred to larger problems.

### Hyperparameter analysis

This section systematically evaluates the impact of key hyperparameters on the performance of the CoRoPINN algorithm. The experiments focus on three core hyperparameters: the cognitive difficulty coefficient $\beta_{c}$, the initial region size *r*_0_, and the warm-up period *T warmup*. These parameters collectively influence the dynamics and generalization capability of CoRoPINN. The sensitivity and optimal ranges of each parameter were assessed using various partial differential equations, with all results documented based on the conventional PINN model.

#### Initial region size *r*_0_.

The base region radius *r*_0_ is a critical hyperparameter in the CoRoPINN method’s region optimization mechanism. The value of *r*_0_ is directly correlated with the approximation accuracy of Monte Carlo region integration and the model’s generalization capability.

**Fig 8:**
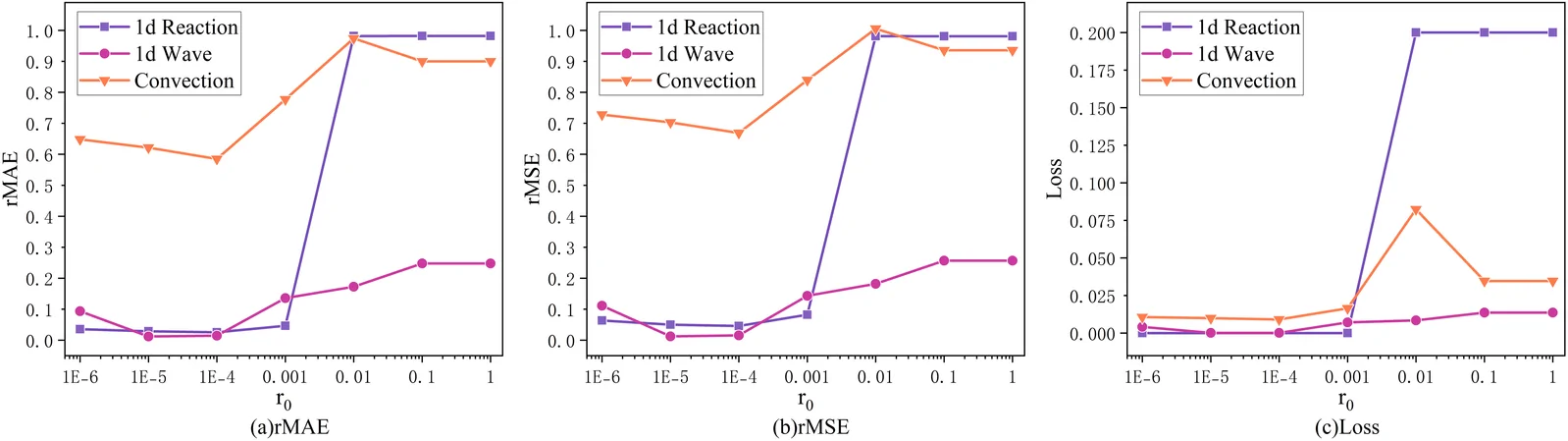
Performance of CoRoPINN on three PDEs using the PINN backbone under different initial region sizes.

Fig 8 shows the rMAE, rMSE, and Loss curves for different values of *r*_0_ across three PDEs. When $r_{0}\leq 10^{-3}$, the three metrics generally improve as *r*_0_ increases, indicating that moderate domain expansion can strengthen the continuity of the physical constraints. When $r_{0}\geq 10^{-2}$, performance generally deteriorates, indicating that an excessively large domain can compromise optimization stability. Based on these evaluated problems, an initial radius in the range $\left[10^{-5},10^{-4}\right]$ is recommended.

#### Cognitive difficulty coefficient $\beta_{c}$.

The cognitive difficulty coefficient $\beta_{c}$ regulates the adaptability of region size to sample difficulty. This parameter directly influences the additional sampling range allocated to challenging samples and serves as a crucial factor in balancing local constraint strength and optimization stability.

**Fig 9:**
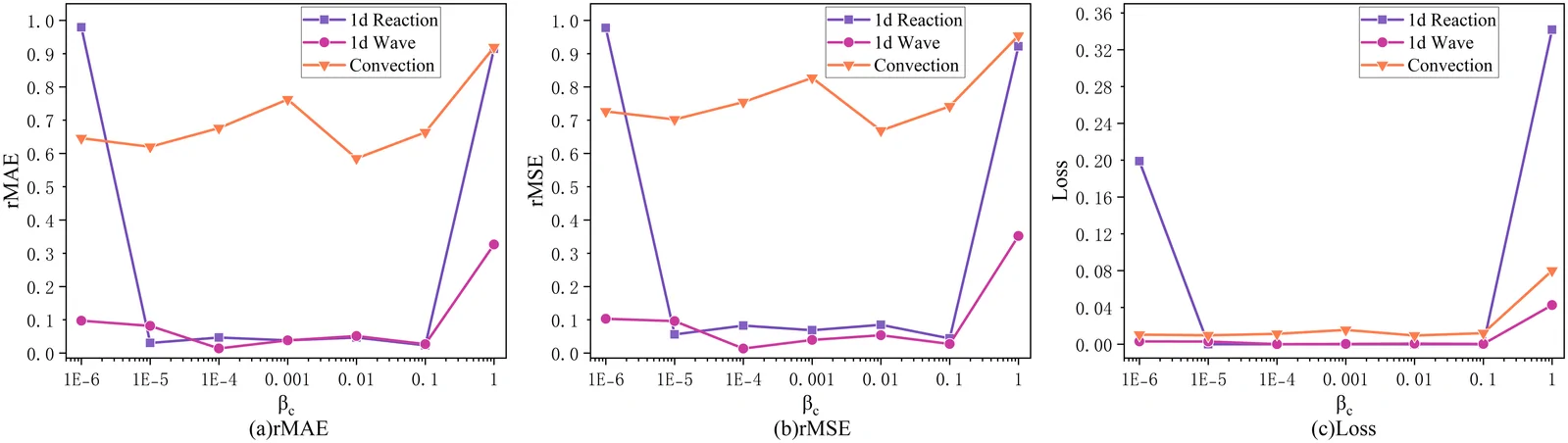
Performance of CoRoPINN on three PDEs using the PINN backbone under different cognitive difficulty coefficients.

Fig 9 evaluates CoRoPINN across three PDEs under different values of $\beta_{c}$. The best overall performance on the evaluated problems occurs when $\beta_{c}$ lies in $\left[10^{-5},10^{-4}\right]$. Within this interval, the cognitive weighting mechanism balances the contributions of easy and difficult samples. Performance deteriorates when $\beta_{c}$ is below 10^−5^ or above 10^−1^. Based on these results, $\beta_{c}$ should be selected within $\left[10^{-5},10^{-1}\right]$ and tuned for the target problem.

#### Warm-up period $T_{warmup}$.

The warm-up period *T warmup* determines when the model begins to focus on challenging samples. An insufficient warm-up period may lead to instability during early training, whereas an excessively long warm-up period can delay learning in important areas such as boundaries.

**Fig 10:**
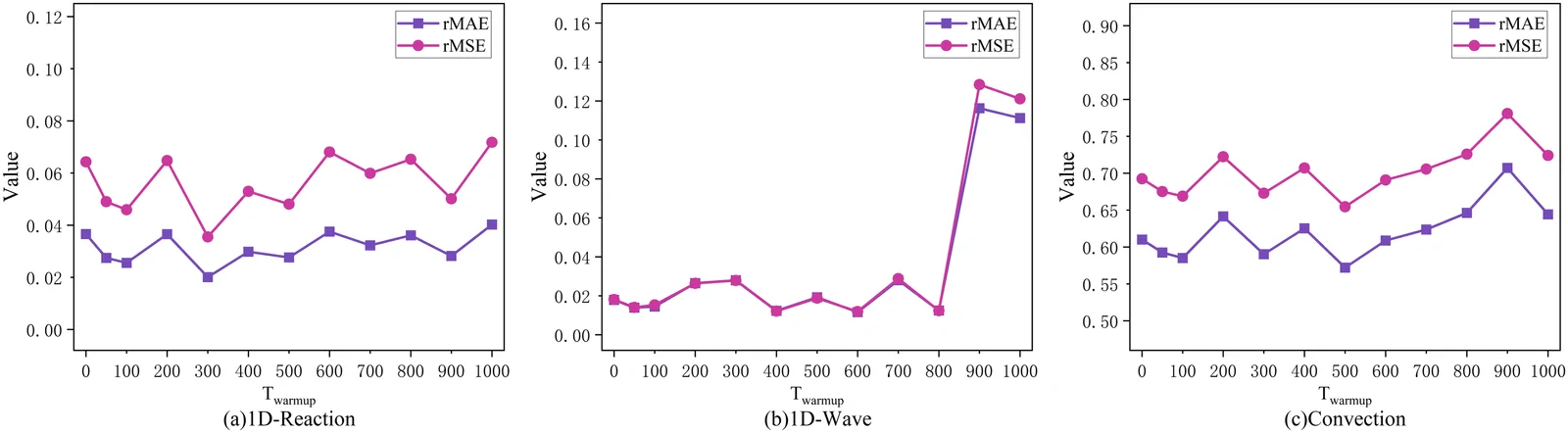
Performance of CoRoPINN on three PDEs using the PINN backbone under different warm-up periods.

Fig 10 shows the performance of CoRoPINN across three PDEs under different values of *T*_warmup_. For 1D-Reaction, rMAE remains between 0.02 and 0.04 across the evaluated range. For 1D-Wave, the model remains stable when *T*_warmup_ is in [0,800], with rMAE and rMSE between 0.01 and 0.03. When *T*_warmup_ reaches 900 or more, rMAE and rMSE increase to 0.11 and 0.13, respectively. For Convection, rMAE and rMSE increase gradually over [0,1000]. Based on these evaluated problems, a practical initial range is [100,600].

## Discussion

### Limitations

The results should be interpreted in light of three limitations. First, gradient-statistic calibration, pointwise difficulty evaluation, and Monte Carlo region sampling add computation beyond standard PINN training. Table 5 quantifies this trade-off under a common setup and shows that its magnitude depends on the PDE and comparator. Second, performance depends on the initial region radius *r*_0_, cognitive difficulty coefficient $\beta_{c}$, and warm-up period *T*_warmup_. Figs 8–Fig 9, Fig 10 identify useful ranges for the evaluated problems, but these ranges may require retuning for previously unseen PDEs or backbone architectures [43]. Third, although the experiments cover one-dimensional time-dependent equations, Poisson problems up to five dimensions, and one two-dimensional nonlinear coupled Burgers problem, they do not establish performance for arbitrary high-dimensional domains, complex geometries, or broader coupled multiphysics systems. These boundaries limit the extent to which the present results can be generalized.

### Future work

Future work will address these limitations in three directions. More efficient reuse of gradient statistics and adaptive allocation of region samples may reduce the additional training cost. Joint or automatic selection of *r*_0_, $\beta_{c}$, and *T*_warmup_ may improve transfer across PDEs while reducing manual tuning. Further evaluation should extend CoRoPINN to higher-dimensional equations, complex geometries, broader coupled and multiphysics systems, and a wider range of backbone architectures. The Poisson-5D and Burgers2D experiments provide initial tests of high-dimensional and nonlinear coupled settings, respectively, but broader validation is needed to determine the limits of these extensions.

## Conclusion

This paper presents CoRoPINN, a cognition-guided region optimization method that couples the spatial continuity of RoPINN with the progressive difficulty scheduling of CoPINN. Rather than placing the two mechanisms side by side, CoRoPINN forms a closed feedback loop: gradient-stability statistics calibrate a globally stable radius, PDE residuals estimate pointwise difficulty, and this difficulty jointly controls adaptive neighborhoods and cognitive weights. The resulting optimization progressively redirects learning toward difficult regions while maintaining continuous physical constraints around collocation points.

Across the evaluated 1D-Reaction, 1D-Wave, and Convection problems, CoRoPINN obtains the lowest geometric-mean rMAE over all five backbone architectures, reducing the aggregate error relative to the second-best method by 21.0%, 61.6%, and 52.9%, respectively. In the multidimensional tests, CoRoPINN ranks first in both metrics for 2D and 5D Poisson and second in 3D. It also achieves the lowest rMAE and rMSE among all four PINN-based methods on Burgers2D. Taken together, these results show that CoRoPINN’s main advantage lies in using the same residual-derived difficulty signal to decide both where continuous constraints should be expanded and where optimization effort should be concentrated. This coordinated adaptation makes more effective use of sparse collocation information while preserving neighborhood continuity, allowing the method to retain strong performance across changes in backbone, dimensionality, and PDE dynamics. Within the evaluated scope, CoRoPINN is the strongest overall method among the compared baselines.
